# Bullying in Physical Education During Compulsory Primary Education: Prevalence, Typologies and Differences by Sex and Year Group

**DOI:** 10.3390/children13070970

**Published:** 2026-07-22

**Authors:** Rafael Clavero-Prados, Flavia Estefanía Amar-Cantos, Javier Murillo-Moraño, José Manuel Armada-Crespo, Álvaro Morente-Montero, Juan de Dios Benítez-Sillero

**Affiliations:** 1 Research Group in Sport and Physical Education for Personal and Social Development (GIDEPSO), Department of Specific Didactics, University of Córdoba, 14071 Cordoba, Spain; z42clprr@uco.es (R.C.-P.); m62arcrj@uco.es (J.M.A.-C.); eo1momoa@uco.es (Á.M.-M.); eo1besij@uco.es (J.d.D.B.-S.); 2 Research Group in Sport and Physical Education for Personal and Social Development (GIDEPSO), Faculty of Education Sciences, University of Cádiz, 11519 Cádiz, Spain; flavia.amar@uca.es; 3 Research Group in Sport and Physical Education for Personal and Social Development (GIDEPSO), Teacher Training College “Sagrado Corazón”, University of Córdoba, 14006 Córdoba, Spain

**Keywords:** bullying, physical education, compulsory primary education, victim, perpetrator

## Abstract

**Highlights:**

**What are the main findings?**

- Social exclusion was the most frequent form of bullying during PE lessons in primary school.
- Bully-victim roles were more common in the lower primary school years.

**What are the implications of the main findings?**

- Inclusive group formation and cooperative learning should be prioritised to prevent bullying in PE.
- Early identification and intervention should be prioritised in the first years of primary school.

**Abstract:**

**Background/Objectives:** Bullying represents a serious threat to the physical, psychological, and social well-being of students, with particular relevance in Physical Education (PE) contexts, where the physical and competitive nature of the setting may facilitate specific forms of aggression. Despite growing research interest, evidence on the prevalence and distribution of bullying roles and typologies within PE remains limited, particularly in primary education. This study aimed to analyse the prevalence of bullying victimisation, perpetration, and bully-victim profiles among primary school pupils in PE, as well as to examine differences by sex and year group across the different forms of bullying behaviour. **Methods:** A cross-sectional study was conducted with an analytical sample of 831 pupils from Year 3 to Year 6 of compulsory primary education in Córdoba, Spain. Bullying roles and typologies were assessed using a validated self-report instrument. Differences by sex and year group were examined through appropriate inferential analyses. **Results:** A total of 21.1% of pupils were identified as victims, 5% as perpetrators, and 8.5% as bully-victims. The most frequent form of perpetration was exclusion/rejection (7.9%), surpassing physical and verbal aggression. Boys showed greater physical victimisation and a higher tendency towards the pure perpetrator role, whilst exclusion showed no significant differences by sex. Physical and verbal behaviours decreased as year group advanced, whereas exclusion remained stable throughout the entire primary stage. The bully-victim profile was more prevalent in lower year groups. **Conclusions:** The findings highlight the particular relevance of exclusion/rejection as the predominant form of bullying in PE, which persists across year groups regardless of sex. These results underscore the need to design inclusive groupings, promote cooperative tasks, and implement early intervention strategies. Specific training for PE teachers in bullying detection and intervention is identified as a priority to address this phenomenon effectively within the PE context.

## 1. Introduction

Bullying is defined as a form of intentional interpersonal aggression, repeated over time and characterised by a power imbalance between the aggressor or aggressors and the victim [1,2]. This widely accepted definition in the scientific literature distinguishes bullying from isolated peer conflicts and underscores the sustained and asymmetric nature of the behaviours involved [3]. Bullying may manifest in three main forms: direct physical aggression (hitting, pushing), verbal aggression (insults, mockery, ridicule), and social exclusion or rejection (isolation from the group, ostracism, rumour spreading) [4,5].

Prevalence rates of school bullying vary depending on the methodology used, the measurement instrument, and the context studied, ranging from 10% to 35% of students involved in some role [5,6]. The consequences of bullying are far-reaching for all those involved: victims exhibit lower academic performance, mental health problems, sleep disorders, and, in the most severe cases, suicidal ideation [7]; perpetrators show a heightened risk of long-term antisocial behaviour; and the most vulnerable profile is that of the bully-victim, who accumulates the negative effects of both roles and presents the poorest indicators of psychosocial adjustment [8,9]. Sex differences in bullying have also been widely documented. Previous research has consistently shown that boys are more likely to be involved in direct forms of bullying, particularly physical aggression, whereas girls tend to engage more frequently in relational forms, although these differences are not always consistent across contexts [10,11]. Recent evidence also suggests that patterns of bullying involvement may vary according to the role adopted, with boys more frequently represented among perpetrators and bully-victims, while girls are more commonly found in the uninvolved group or as victims of indirect forms of aggression [12]. With regard to age-related trends, research indicates that bullying tends to be most frequent in the final years of primary school and the first years of secondary school, decreasing progressively as schooling advances [13,14,15]. This developmental pattern has also been observed in the Spanish context, where cross-sectional and longitudinal studies document differences by school year in bullying involvement, although the trend is not always linear and may vary according to the role considered [11].

The Physical Education (PE) context presents characteristics that make it a particularly relevant setting for the study of school bullying. Greater freedom of movement, direct physical interaction, the regular presence of competitive activities, group and team formation, and reduced teacher supervision in spaces such as changing rooms or outdoor areas create conditions that may facilitate bullying behaviour [16,17]. Furthermore, in PE, physical ability, body image, and sporting performance acquire a visibility that has no equivalent in other school subjects, which may amplify dynamics of humiliation, mockery, or exclusion towards pupils with lower motor competence or different body types [18]. A systematic review [17] focused specifically on the correlates of bullying in PE confirms that individual, relational, and contextual factors interact in complex ways within this setting, with teaching style and group dynamics emerging as primary moderating variables.

Nevertheless, Physical Education (PE) also offers unique conditions that make it a privileged context for bullying prevention. Beyond promoting cognitive development, PE supports affective, psychomotor, and social growth [19], providing opportunities to foster cooperation, inclusion, empathy, and positive peer relationships through appropriately designed learning experiences. When teachers design cooperative learning situations, promote respectful relationships, actively manage conflict, and ensure the inclusion of all pupils in activities, PE lessons can act as a protective factor against bullying dynamics within the school [20,21]. This dual nature (risk and opportunity) has been highlighted by several studies in the Spanish context: [22] found lower perpetration rates in PE than in the general school setting among secondary school students, with significantly higher perpetration among boys; and [23] observed that the large majority of students identified as victims or perpetrators in the general school context were no longer identified as such in PE lessons, pointing to the potential of well-managed PE to deactivate conflict. In Poland, Ref. [24] found that perceived peer support during PE lessons significantly moderated involvement in bullying roles. Specific training for PE teachers in bullying detection and intervention therefore emerges as an urgent need [25].

Despite this growing body of interest, research on bullying in PE has focused almost exclusively on secondary education [22,24,26,27], using varying bullying assessment instruments, which makes direct comparisons difficult. The primary school stage and, in particular, Years 3 to 6 of compulsory primary education have not been studied in this subject area, despite bullying rates generally being higher in this age range than in secondary school [28,29]. Accordingly, the aim of this study is to analyse the prevalence and incidence of school bullying in the PE context among pupils in Years 3 to 6 of compulsory primary education, examining differences by sex and school year, using a questionnaire adapted for this age group.

## 2. Materials and Methods

### 2.1. Design and Participants

A descriptive cross-sectional study was conducted using non-probability convenience sampling. A total of *N* = 842 pupils were initially recruited. Of these, 6 participants who identified with another gender option and 5 participants with unreported sex were excluded from all analyses, the former due to insufficient cell size to support statistically meaningful comparisons and the latter because sex could not be determined, yielding a final analytic sample of *N* = 831 (Mage = 10.25 years, SD = 1.19, range = 7.92–13.38; 393 girls, Mage = 10.23, SD = 1.17; and 438 boys, Mage = 10.27, SD = 1.21), distributed across four year groups of compulsory primary education: Year 3 (*n* = 137, Mage = 8.49, SD = 0.38), Year 4 (*n* = 229, Mage = 9.53, SD = 0.40), Year 5 (*n* = 198, Mage = 10.53, SD = 0.41), and Year 6 (*n* = 267, Mage = 11.57, SD = 0.48). The 10 participating schools are located in the province of Córdoba (Andalusia, Spain) and were invited to participate based on their willingness to collaborate with the research team and their accessibility. The inclusion criteria were (a) being enrolled in Years 3 to 6 of Primary Education, (b) attending one of the participating schools, and (c) providing written informed consent from parents or legal guardians together with the student’s assent. The study was conducted in accordance with the ethical principles set out in the Declaration of Helsinki, with authorisation from the schools’ management teams and informed consent from the families of participating pupils. The anonymity, confidentiality, and voluntary nature of participation were guaranteed at all times.

### 2.2. Instrument

School bullying in the PE context was measured using a questionnaire with two principal dimensions: victimisation and perpetration. The instrument is based on the questionnaire developed by [30], which has demonstrated structural validity for measuring both dimensions of school bullying, and follows the adaptation to the specific PE context carried out by [24] in their study with Polish adolescents. The questionnaire assesses three types of behaviour: physical aggression (hitting/pushing), verbal aggression (insults/mockery), and social exclusion/rejection. To enhance the sensitivity of the instrument, the response options were extended to five levels (0 = has not happened in the past two months; 1 = has only happened once or twice; 2 = two or three times a month; 3 = approximately once a week; 4 = several times a week), following the frequency-scale model used in validated instruments in the Spanish context such as the EBIP-Q [1]. Following previous research on school bullying [1,28], a pupil was considered to be involved in a bullying role when reporting a frequency of ≥2 (“two or three times a month”) on at least one victimisation or perpetration item. This threshold is consistent with the widely accepted operational definition of bullying, which requires repeated rather than isolated aggressive behaviours. Given the modification of the response options to five ordered categories, the items were treated as ordinal rather than continuous. The model was estimated in R (v. 4.5.2; R Foundation for Statistical Computing, Vienna, Austria) using the lavaan package (v. 0.6-21; Rosseel, Ghent, Belgium), with the robust weighted least squares estimator with mean- and variance-adjusted test statistic (WLSMV), the estimator recommended for ordinal indicators, based on polychoric correlations among items. The model was specified as two correlated factors, Perpetration and Victimisation, each defined by three indicators (hitting/pushing, insults/mockery, exclusion/rejection), with latent variances fixed to 1 for identification (Delta parameterisation) and the two factors allowed to covary freely. Because full-information maximum likelihood (FIML) is not available for categorical/WLSMV estimation, missing data were handled using pairwise present-data deletion, the default and recommended approach for WLSMV models. Of the *N* = 831 participants in the analytic sample, 4 were excluded automatically for having no valid response on any of the six items, yielding an effective estimation sample of *n* = 827 (pairwise coverage ranged from 99.4% to 99.9% across item pairs). The model showed an acceptable fit to the data: χ^2^(8) = 34.539, *p* < 0.001 (scaling correction factor = 0.652); CFI = 0.977; TLI = 0.957; RMSEA = 0.063, 90% CI [0.043, 0.086], *p*(RMSEA ≤ 0.05) = 0.136; SRMR = 0.047. All factor loadings were statistically significant (*p* < 0.001). For the ‘Perpetration’ factor, standardised estimates ranged from λ = 0.574 to λ = 0.747; for the ‘Victimisation’ factor, they ranged from λ = 0.661 to λ = 0.818. The standardised covariance between the two factors was 0.657 (*p* < 0.001), suggesting a moderate-to-strong relationship between the dimensions analysed. Internal consistency was α = 0.612 (ω = 0.585) for perpetration and α = 0.697 (ω = 0.690) for victimisation.

### 2.3. Data Analysis

A descriptive analysis of prevalence was conducted using frequencies and percentages based on valid cases for each variable. To examine the association between involvement in bullying behaviours and the variables sex and school year group, Pearson’s chi-square tests (χ^2^) were applied, together with Cramér’s V as a measure of effect size. For the analyses involving the bullying-role variable (uninvolved, victim only, perpetrator only, bully-victim), adjusted standardised residuals were additionally examined at the cell level, both when the overall χ^2^ was significant, to identify which specific categories drove the association, and when it was not, as an exploratory check for isolated cell-level differences (|z| > 1.96). Pupils were classified into four categories: 0 = uninvolved; 1 = victim only; 2 = perpetrator only; 3 = bully-victim. All descriptive and inferential analyses were carried out on valid cases for each variable using Python (version 3.14.5; Python Software Foundation, Wilmington, DE, USA).

## 3. Result

### 3.1. Missing Data

The percentage of missing values ranged between 0.6% and 1.1% of the total sample (Table 1). Given their small magnitude and random distribution, analyses were conducted on the available valid cases for each variable, without imputation.

### 3.2. Prevalence of Bullying in Physical Education

The most frequent form of victimisation was receiving insults/mockery (*n* = 140; 17.0%), followed by exclusion/rejection as a victim (*n* = 129; 15.7%) and hitting/pushing as a victim (*n* = 113; 13.7%). Regarding the perpetrator role, the most prevalent behaviour was exclusion/rejection (*n* = 65; 7.9%), followed by hitting/pushing (*n* = 48; 5.8%) and insulting/ridiculing (*n* = 35; 4.2%) (Table 2).

### 3.3. Differences by Sex

χ^2^ analyses (2 × 2, df = 1) for each type of behaviour showed statistically significant differences in perpetration by hitting/pushing, with higher prevalence among boys (7.4% vs. 4.1%; χ^2^(1) = 4.15, *p* = 0.042), and in victimisation by hitting/pushing (16.9% vs. 10.2%; χ^2^(1) = 7.94, *p* = 0.005) and by insults/mockery (20.0% vs. 13.7%; χ^2^(1) = 5.63, *p* = 0.018), both higher among boys. Perpetration by insults approached the significance threshold (χ^2^(1) = 3.82, *p* = 0.051), also higher among boys. Exclusion/rejection showed no significant association with sex in either role (Table 3). However, effect sizes (Cramér’s V) for all these associations were negligible to small (V = 0.026–0.098), indicating that, despite reaching statistical significance, the practical magnitude of the sex differences was limited.

Regarding the bullying role, 65.6% of pupils (*n* = 539) showed no involvement in any bullying behaviour in PE, 20.9% (*n* = 172) were identified exclusively as victims, 5.0% (*n* = 41) exclusively as perpetrators, and 8.5% (*n* = 70) as bully-victims.

Analysis of the bullying role by sex did not yield a statistically significant overall association [χ^2^(3) = 6.56, *p* = 0.087, V = 0.089; valid *N* = 822]. Given the non-significant omnibus test, the cell-level residuals reported in Table 4 should be interpreted with caution and are presented for exploratory purposes only. With this caveat, girls showed a residual above the conventional threshold in the uninvolved role (z = +2.05) and boys in the pure-perpetrator role (z = +2.10); however, as the overall association was not significant, these isolated patterns should be regarded as hypothesis-generating and would require confirmation in future, larger samples rather than substantive interpretation at this stage. The victim-only (z = −0.86/+0.86) and bully-victim roles (z = −0.60/+0.60) did not present residuals exceeding the |z| > 1.96 threshold (Table 4).

### 3.4. Differences by Year Group

Victimisation by hitting/pushing showed the strongest association with school year group [χ^2^(3) = 22.48, *p* < 0.001, V = 0.165], decreasing from 24.3% in Year 3 to 7.2% in Year 6. Verbal victimisation by insults/mockery was also significant [χ^2^(3) = 13.12, *p* = 0.004, V = 0.126], declining from 25.7% to 11.7%. Perpetration of hitting/pushing decreased significantly [χ^2^(3) = 12.39, *p* = 0.006, V = 0.122], from 11.0% to 2.6%. Exclusion behaviours, both as perpetrator and as victim, showed no significant association with year group (*p* = 0.685 and *p* = 0.170, respectively), and insults/mockery as perpetrator was also non-significant (*p* = 0.227) (Table 5). As with the sex comparisons, all effect sizes remained in the small range (V = 0.042–0.165), indicating that year-group differences, while statistically detectable, were modest in practical magnitude.

Analysis of the bullying role by year group revealed a statistically significant association [χ^2^(9) = 23.85, *p* = 0.005, V = 0.098]. The uninvolved group increased progressively from 55.1% (*n* = 75) in Year 3 to 71.6% (*n* = 189) in Year 6 (adjusted residuals: z = −2.80 in Year 3, z = +2.50 in Year 6), whilst the bully-victim profile decreased from 13.2% (*n* = 18) to 4.9% (*n* = 13) (z = +2.16 in Year 3, z = −2.54 in Year 6). The victim-only role was also significantly over-represented in Year 3 (z = +2.66) and under-represented in Year 6 (z = −1.88) (Table 6).

## 4. Discussion

The findings of this study provide evidence on the prevalence of bullying in the specific context of PE during primary education, contributing to filling a significant gap in the scientific literature. The results demonstrate that bullying in PE is present from the earliest years of primary school, with direct implications for teaching practice. The rates of victims (≈21%) and perpetrators (5%), as well as bully-victims (8.5%), exceed those reported by [22] in secondary education using the same procedure, albeit with a different instrument, which is consistent with the higher prevalence of bullying at younger ages documented in the literature [28,29]. Comparable figures were obtained in the study by [24], with 19% of victims and 23% of perpetrators in PE lessons; by [27], with 15% of perpetrators and 28% of victims; and by [26], with 30% victimisation and 29% perpetration rates in PE.

The perpetration rate (5%) is, however, substantially lower than the victimisation rate, indicating that bullying in primary PE is characterised by a large group of victims and a relatively small number of active perpetrators, in line with the data reported by [23]. This finding is consistent with recent evidence from elementary physical education settings, which also identified bullying behaviours as an ongoing concern and highlighted the need for systematic monitoring to ensure safe and inclusive learning environments [31].

The most pedagogically significant finding is that exclusion/rejection is the most frequent form of perpetration in PE (7.9%), above physical (5.9%) and verbal (4.3%) aggression. This indicates that the predominant mechanism of bullying in PE lessons is not hitting or insults, but rather rejection from groups, exclusion from teams, or isolation during activities. Accordingly, didactic design emerges as a key intervention variable: when teachers manage group formation, design cooperative tasks, and actively supervise inclusion dynamics, they are addressing the most prevalent vector of bullying in their subject [20]. Moreover, this practical implication is reinforced by recent evidence showing that PE teachers identify classroom management, lesson organisation, and specific training in bullying prevention as essential elements for effectively addressing bullying behaviours during physical education lessons [32]. Conversely, when PE is organised around free group formation or unmanaged competition, it may amplify pre-existing exclusion dynamics within the class group [23], a finding consistent with the characterisation of PE as a space for conflict deactivation only when adequately managed.

Regarding sex differences in types of bullying behaviour, the higher rate of physical victimisation among boys is consistent with the accumulated evidence in the literature [10,11]. Analysis of bullying roles adds an important nuance: although the overall chi-square was not statistically significant (*p* = 0.243), standardised residuals indicate that girls are over-represented in the uninvolved group (z = +2.1) and boys in the pure perpetrator role (z = +2.3). This result, consistent with the gender profiles described by [17], suggests that, in the PE context, boys are not only more frequently victimised physically, but also engage in aggression in a more unidirectional manner. Exclusion, however, showed no significant sex differences, pointing to a more equitable distribution of this form of relational bullying in the physical sport context, unlike findings from other settings where girls show higher rates of relational bullying [33].

Lower prevalence rates of physical victimisation, verbal victimisation, and the bully-victim role were observed in the higher year groups. These findings are consistent with previous research reporting differences in bullying involvement across educational stages [14,15]. Although developmental models suggest that age-related improvements in emotional and social self-regulation may contribute to changes in bullying behaviours [13], the cross-sectional design of the present study does not allow these differences to be interpreted as evidence of individual developmental change over time. Rather, they reflect differences between pupils from different year groups assessed at a single point in time. In contrast, exclusion behaviours remained relatively stable across all year groups, suggesting that this form of bullying warrants continued attention throughout primary education. Moreover, the higher prevalence of the bully-victim role observed in the lower year groups reinforces the importance of implementing preventive strategies from the early years of primary education, given the greater vulnerability associated with this profile [8,9].

As limitations of the study, it should be noted that the cross-sectional design does not allow causal relationships to be established, nor does it permit the tracking of individual changes in bullying roles over time. The use of non-probability sampling limits the generalisability of findings beyond the studied sample, and the geographical restriction to schools in the province of Córdoba further constrains external validity. In addition, the perpetration scale showed relatively modest internal consistency (α = 0.612; ω = 0.585), which should be considered when interpreting the findings. This may be related to the small number of items included in the scale and the adaptation of the response format to five ordered categories. The exclusive use of self-report measures may also have introduced social desirability bias and possible underreporting of bullying behaviours, particularly in the perpetrator role. Finally, all denominators, valid sample sizes, and percentages were carefully rechecked and corrected throughout the manuscript following a complete verification of the database.

Future research should incorporate longitudinal designs, territorially broader samples, and mediating variables such as teaching style, classroom climate, grouping strategies, and pupils’ socio-emotional competence.

## 5. Conclusions

Bullying in PE is present from the earliest years of primary school, with rates that appear higher than those reported in secondary education. Exclusion is the most frequent form of perpetration in PE, which positions grouping design and cooperative tasks as primary preventive strategies for teachers. Boys show higher rates of physical victimisation and a greater tendency towards the pure perpetrator role in PE. Exclusion, however, shows no significant sex differences, requiring group inclusion strategies that address all pupils regardless of gender. Physical and verbal bullying behaviours were less frequent in the higher year groups, whereas exclusion behaviours remained relatively stable across the entire primary stage. The bully-victim profile is most prevalent in the lower year groups, underscoring the importance of early intervention in PE and of specific teacher training in the detection and management of school bullying.

## Figures and Tables

**Table 1:** Valid and Missing Data by Variable.

Role	Valid *n*	Missing *n*	% Missing
Perpetrator—Hitting	826	5	0.6%
Perpetrator—Insults	826	5	0.6%
Perpetrator—Exclusion	826	5	0.6%
Perpetrator—Overall (composite)	826	5	0.6%
Victim—Hitting	824	7	0.8%
Victim—Insults	824	7	0.8%
Victim—Exclusion	823	8	1.0%
Victim—Overall (composite)	823	8	1.0%
Bullying Role	822	9	1.1%

Note. *N* = 831 (six participants who selected “other” for sex and five with unreported sex were excluded from all analyses). Missing values correspond to unanswered items. The “Overall (composite)” perpetrator/victim variables require all three items of the corresponding block to be answered; a participant missing any single item within a block is treated as missing on the composite. The Bullying Role variable requires both composites to be valid and is therefore missing for the union of participants missing either block (*n* = 9).

**Table 2:** Prevalence of Bullying Behaviours in PE by Role and Type.

Behaviour	Role	*n* No (%)	*n* Yes (%)	*n* Valid
Hitting/pushing	Perpetrator	778 (94.2%)	48 (5.8%)	826
Insults/mockery	Perpetrator	791 (95.8%)	35 (4.2%)	826
Exclusion/rejection	Perpetrator	761 (92.1%)	65 (7.9%)	826
Hitting/pushing	Victim	711 (86.3%)	113 (13.7%)	824
Insults/mockery	Victim	684 (83.0%)	140 (17.0%)	824
Exclusion/rejection	Victim	694 (84.3%)	129 (15.7%)	823

Note. Percentages are calculated on valid cases for each variable.

**Table 3:** Prevalence of Bullying Behaviours in PE by Sex and Chi-Square Tests.

Behaviour	Role	Girls *n* (%)	Boys *n* (%)	χ^2^ (df)	*p*	Cramér’s V
Hitting/pushing	Perpetrator	16 (4.1%)	32 (7.4%)	4.15 (1)	0.042 *	0.071
Insults/mockery	Perpetrator	11 (2.8%)	24 (5.5%)	3.82 (1)	0.051	0.068
Exclusion/rejection	Perpetrator	28 (7.1%)	37 (8.5%)	0.57 (1)	0.449	0.026
Hitting/pushing	Victim	40 (10.2%)	73 (16.9%)	7.94 (1)	0.005 *	0.098
Insults/mockery	Victim	54 (13.7%)	86 (20.0%)	5.63 (1)	0.018 *	0.083
Exclusion/rejection	Victim	68 (17.3%)	61 (14.2%)	1.58 (1)	0.208	0.044

Note. Percentages calculated within each sex group on valid cases. * *p* < 0.05. Participants who selected “other” (*n* = 6) were excluded entirely from these analyses. Cramér’s V interpretation (df = 1): <0.10 negligible, 0.10–0.30 small, 0.30–0.50 medium, >0.50 large.

**Table 4:** Distribution of Bullying Role by Sex with Adjusted Standardised Residuals.

Role	Description	Girls *n* (%)	z	Boys *n* (%)	z	Total *n* (%)
0	Uninvolved	271 (69.1%)	2.05 *	268 (62.3%)	−2.05 *	539 (65.6%)
1	Victim only	77 (19.6%)	−0.86	95 (22.1%)	0.86	172 (20.9%)
2	Perpetrator only	13 (3.3%)	−2.10 *	28 (6.5%)	2.10 *	41 (5.0%)
3	Bully-victim	31 (7.9%)	−0.60	39 (9.1%)	0.60	70 (8.5%)
—	Total	392 (100%)	—	430 (100%)	—	822 (100%)

Note. Valid *N* = 822. * |z| > 1.96. χ^2^(3) = 6.56, *p* = 0.087, Cramér’s V = 0.089 (overall non-significant).

**Table 5:** Prevalence of Bullying Behaviours in PE by Year Group and Chi-Square Tests.

Behaviour	Role	3rd *n* (%)	4th *n* (%)	5th *n* (%)	6th *n* (%)	χ^2^ (df)	*p*	Cramér’s V
Hitting/pushing	Perpetrator	15 (11.0%)	16 (7.0%)	10 (5.1%)	7 (2.6%)	12.39 (3)	0.006 *	0.122
Insults/mockery	Perpetrator	9 (6.6%)	12 (5.2%)	7 (3.6%)	7 (2.6%)	4.34 (3)	0.227	0.073
Exclusion/rejection	Perpetrator	8 (5.9%)	21 (9.2%)	14 (7.1%)	22 (8.3%)	1.49 (3)	0.685	0.042
Hitting/pushing	Victim	33 (24.3%)	32 (14.0%)	29 (14.8%)	19 (7.2%)	22.48 (3)	<0.001 **	0.165
Insults/mockery	Victim	35 (25.7%)	37 (16.2%)	37 (18.9%)	31 (11.7%)	13.12 (3)	0.004 *	0.126
Exclusion/rejection	Victim	26 (19.1%)	40 (17.6%)	32 (16.3%)	31 (11.7%)	5.02 (3)	0.170	0.078

Note. Percentages calculated within each year group on valid cases. * *p* < 0.05; ** *p* < 0.001. Cramér’s V interpretation (df = 1): <0.10 negligible, 0.10–0.30 small, 0.30–0.50 medium, >0.50 large.

**Table 6:** Distribution of Bullying Role by Year Group.

Role	Description	3rd *n* (%)	z	4th *n* (%)	z	5th *n* (%)	z	6th *n* (%)	z	Total *n* (%)
0	Uninvolved	75 (55.1%)	−2.80 *	148 (65.2%)	−0.14	127 (65.1%)	−0.15	189 (71.6%)	2.50 *	539 (65.6%)
1	Victim only	40 (29.4%)	2.66 *	43 (18.9%)	−0.86	44 (22.6%)	0.64	45 (17.0%)	−1.88	172 (20.9%)
2	Perpetrator only	3 (2.2%)	−1.63	11 (4.8%)	−0.12	10 (5.1%)	0.10	17 (6.4%)	1.31	41 (5.0%)
3	Bully-victim	18 (13.2%)	2.16 *	25 (11.0%)	1.58	14 (7.2%)	−0.77	13 (4.9%)	−2.54 *	70 (8.5%)
—	Total	136 (100%)	—	227 (100%)	—	195 (100%)	—	264 (100%)	—	822 (100%)

Note. Valid *N* = 822. * |z| > 1.96. χ^2^(9) = 23.85, *p* = 0.005, Cramér’s V = 0.098.

## Data Availability

The individual-level data presented in this study are available from the corresponding author upon reasonable request, subject to ethical approval and institutional regulations. The data are not publicly available due to privacy and ethical restrictions related to research involving minors. Aggregated data tables and the statistical analysis scripts used in the study may also be provided upon request.
